# The genetic study of three population microisolates in South Tyrol (MICROS): study design and epidemiological perspectives

**DOI:** 10.1186/1471-2350-8-29

**Published:** 2007-06-05

**Authors:** Cristian Pattaro, Fabio Marroni, Alice Riegler, Deborah Mascalzoni, Irene Pichler, Claudia B Volpato, Umberta Dal Cero, Alessandro De Grandi, Clemens Egger, Agatha Eisendle, Christian Fuchsberger, Martin Gögele, Sara Pedrotti, Gerd K Pinggera, Stefan A Stefanov, Florian D Vogl, Christian J Wiedermann, Thomas Meitinger, Peter P Pramstaller

**Affiliations:** 1 Institute of Genetic Medicine, European Academy, Bolzano, Italy; 2 Department of Gynaecology, Hospital of Merano, Via Rossini 5, 39012 Merano-Meran, Italy; 3 Laboratory of Medical Intensive Care, Division of General Internal Medicine, Department of Medicine, Medical University of Innsbruck, Innsbruck, Austria; 4 Division of Internal Medicine II, Department of Medicine, Central Hospital of Bolzano, Bolzano/Bozen, Italy; 5 Institute of Human Genetics, Technical University of Munich, Munich, Germany; 6 GSF – National Research Center for Environment and Health, Institute of Human Genetics, München-Neuherberg, Germany; 7 Department of Neurology, University of Lübeck, Lübeck, Germany; 8 Department of Neurology, General Regional Hospital, Bolzano, Italy

## Abstract

**Background:**

There is increasing evidence of the important role that small, isolated populations could play in finding genes involved in the etiology of diseases. For historical and political reasons, South Tyrol, the northern most Italian region, includes several villages of small dimensions which remained isolated over the centuries.

**Methods:**

The MICROS study is a population-based survey on three small, isolated villages, characterized by: old settlement; small number of founders; high endogamy rates; slow/null population expansion. During the stage-1 (2002/03) genealogical data, screening questionnaires, clinical measurements, blood and urine samples, and DNA were collected for 1175 adult volunteers. Stage-2, concerning trait diagnoses, linkage analysis and association studies, is ongoing. The selection of the traits is being driven by expert clinicians. Preliminary, descriptive statistics were obtained. Power simulations for finding linkage on a quantitative trait locus (QTL) were undertaken.

**Results:**

Starting from participants, genealogies were reconstructed for 50,037 subjects, going back to the early 1600s. Within the last five generations, subjects were clustered in one pedigree of 7049 subjects plus 178 smaller pedigrees (3 to 85 subjects each). A significant probability of familial clustering was assessed for many traits, especially among the cardiovascular, neurological and respiratory traits. Simulations showed that the MICROS pedigree has a substantial power to detect a LOD score ≥ 3 when the QTL specific heritability is ≥ 20%.

**Conclusion:**

The MICROS study is an extensive, ongoing, two-stage survey aimed at characterizing the genetic epidemiology of Mendelian and complex diseases. Our approach, involving different scientific disciplines, is an advantageous strategy to define and to study population isolates. The isolation of the Alpine populations, together with the extensive data collected so far, make the MICROS study a powerful resource for the study of diseases in many fields of medicine. Recent successes and simulation studies give us confidence that our pedigrees can be valuable both in finding new candidates loci and to confirm existing candidate genes.

## Background

The importance of isolated populations for revealing the genetic etiology of common diseases has been frequently highlighted in recent years: the homogeneity of the shared environmental factors and the limited number of recombination events in the DNA make isolates a valuable tool for linkage and association studies [1-5]. Caution was suggested when the isolated population is very large, as in the case of Sardinia and Finland, because the distribution of linkage disequilibrium (LD) across the chromosomes can be similar to that of the general population [6,7]. Nevertheless, when small isolates are concerned, there is increasing evidence of their valuable contribution for finding genes [3,8]. After the difficulties in identifying loci for complex diseases in outbred populations, research on small isolated populations could be a promising way for discovering genotype-phenotype association [9].

Perhaps surprisingly, population isolates are not as rare as it might be expected, and several isolates can be found even within nations of the "old Europe" [10-14], where several population admixture events have taken place over the centuries. Indeed, allele and haplotype distributions across Europe are very differentiated, especially where the Italian peninsula is concerned [15]. Despite their position in the middle of Europe, the Alps seem to be an interesting area for studying genetic isolates [16-18].

Bolzano is the northern-most province of Italy. This province is known as South Tyrol because it is the Southern part of the Tyrol region, split between Italy and Austria. The geographical structure, historical, and political events of this region resulted in the isolation of the population, with scattered villages often located at the ends of remote valleys. Two recent studies, one on the Y-chromosome and mtDNA and autosomal *Alu *markers [19], the other on the extent of LD on Xq13 [20], were designed to assess the homogeneity of the genetic background of isolated groups in South Tyrol. Beyond the ethnic and linguistic differences, in some cases (the Ladin valleys), authors found genetic heterogeneity even between valleys of the same ethnic group, as confirmed by phylogenetic analysis. The expression "*microisolate*" was used to distinguish small subpopulations in remote, high valleys that have conserved an even higher degree of isolation than the cultural and linguistic South Tyrolean isolate, in which they are included [21].

As a part of a larger, ongoing genetic healthcare research program on the South Tyrolean population (the GenNova project), in 2002–2003, an extensive survey (the MICROS study) was carried out on the populations of Stelvio, Vallelunga and Martello, three villages of the Val Venosta. Among them, Stelvio was already classified as a genetic microisolate [20]. The objective of the survey was to screen the population for the presence of several traits by collecting questionnaire-based information, pedigree structure, clinical measurements and blood samples for analyses. The study was intended as the first step of a comprehensive approach for the assessment of the genetics of diseases affecting the South Tyrolean population.

The present article is intended to introduce the MICROS study to the scientific community. We report some preliminary results, and future perspectives are discussed.

## Methods

### Selection of the microisolates

**Figure 1:**
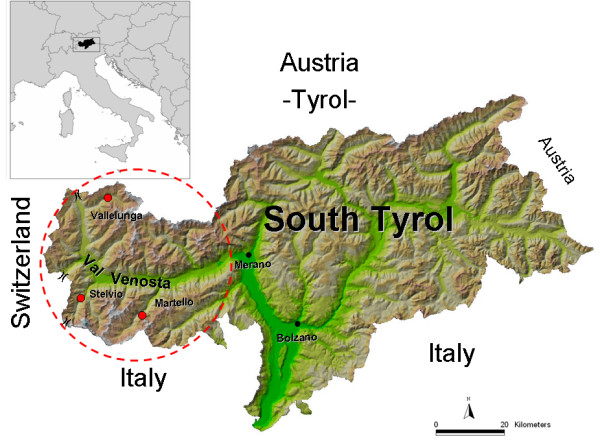
Geographical location of the three microisolates with respect to Val Venosta, South Tyrol, and Europe. Val Venosta, indicated by the red circle, is a 75 km long valley extending west of Merano. The symbols ")(" indicate the most important Alpine passes connecting Val Venosta with other regions.

The villages of Stelvio, Vallelunga and Martello were selected for their geographical and historical isolation and for the presence of highly collaborative local general practitioners (GPs). At the time of the survey, the adult population (aged 18+ year old) numbered 1043 people in Stelvio, 693 in Martell, and 339 in Vallelunga [22]. The geographical location of the villages is presented in Figure 1.

The three villages were selected for study on the basis of four criteria for isolation: i) evidence of an old settlement; ii) a small number of founders; iii) high endogamy rates; iv) slow or null population expansion and negligible immigration. Of course, for verifying these criteria, another prerequisite was the availability and accessibility of historical documentation.

Historical and demographic data on the evolution of the Val Venosta verify that these regions satisfy the criteria for isolation. Numerous archeological findings testify to a broader human presence in the Alpine zone of South Tyrol during the Mesolithic (10,000 years ago) [23]. In 1991 the mummy of a hunter, the so called "Iceman", was discovered on a glacier of the Val Venosta: it was dated to 3300 BC [24]. Documented history of the area began with the Roman conquest in 15 BC. The Latin invaders mixed themselves up with the local tribes, the Raetians, an Alpine population thatstood under influence of Etruscan culture. After the breakup of the Roman Empire in 476 AD, tribes from Northern Europe moved to Italy ("barbaric invasions"); among them, Langobards, Bajuwars, and Alamanni settled in South Tyrol. A period of intense germanization took place during the high middle ages (1000–1300 AD), peaking with the annexation of the county of Tyrol to the Austrian Empire in 1363 [25].

During this period the permanent settlement of the Val Venosta villages occurred, although there is evidence that the remote valleys were known and used for hunting and gathering already by the Raetians. The village of Stelvio represents a partial exception, as there is evidence of settlement and mining activity as early as the Bronze Age (2200-900 B.C.). Nevertheless, even in Stelvio the main expansion took place within the process of enlargement of the cropland, favored by the various landlords. Medieval documents identify a very restrict number of family names accounting for the entire population; some of them are still in use today [26].

Major population bottleneck events recorded were the plague epidemics in Europe during the 14^th ^and 16^th ^century [27,28]. The introduction of church records (Martello and Vallelunga in 1636; Stelvio in 1642) testifies to the presence of new settlers in the 17^th ^century. Some of these were farmers from the surroundings, but in Stelvio and Martello they were mostly mine workers from nearby Switzerland and northern Tyrol. This was the last wave of immigration to these villages before a period of prolonged emigration, caused by the severe poverty, started in the 19^th ^century. People followed different migration patterns and moved to the nearby towns and cities, to other European countries, and to the United States [29]. From the first decade of the 1800s the population was stable or slightly decreasing [30,31]. The high fertility rate of nearly six children per woman counterbalanced the emigration from the valleys. A temporary increase in the population size was observed only for Stelvio, due to the development of tourism in the villages of Solda and Trafoi at the end of the 19^th ^century, before a steep decline in population began. The last big emigration occurred during the period under fascist rule that followed South Tyrol's annexation to Italy in 1919 [32]. The presence of Italian speakers in the villages has always been very limited: after the last census (2001) it was 0.70% of the total population in Martello, 2.33% in the municipality of Curon (which includes Vallelunga) and 2.26% in Stelvio [31].

Analysis of the marriage behavior during the past centuries reveals a strong tendency to endogamy together with a significant percentage of consanguineous unions. However, it should be noted that the vast majority of consanguineous marriages occurred between distant relatives, and that an avoidance of close relatives' marriage has been observed [33]. The marriage behavior did change only with the improvement of the transportation facilities after World War II. Nevertheless, most of the exogamous marriage partners are from the Val Venosta even today (i.e.: no admixture with Italian speaking populations).

Isolation still plays a role in these communities. Mining is no longer an industry in South Tyrol, and these villages have no economic inducements for immigration. Tourism is the leading resource and agriculture is declining. Population is stable or slightly decreasing [22], and very limited admixture seems to be occurred.

The study of the history of Val Venosta population across the centuries suggested that the Val Venosta area could be classified as a *secondary isolate*, that is an isolate that, after being detached from a larger population, "*derived from a relatively small population sample, which then slowly expands, with very little recruitment from outside the group*" [34]. Within this region, we are studying in detail three isolated units, named *microisolates*.

### MICROS-stage 1. Subject enrollment, study setting and data collection

Inhabitants of the target villages were invited, by public advertisement (posters, newspapers, flyers), to attend a meeting with the community mayor, the local GPs, and the scientists leading MICROS. The meeting was designed to give the population a clear understanding of the motivations and importance of MICROS, and to outline how the study would be performed, including the risks and possible benefits to research participants and the community. It was stressed in the meeting that participation was to be on a purely voluntary basis. During the event, the field team (a trained group comprising an expert physician, a nurse, and one to three graduating medical students) was introduced to the population. A call center was established for scheduling appointments. During the appointment, the study was explained by a physician, and a detailed information sheet and a form for the written informed consent were provided to each prospective participant. The information sheet gave details regarding the aims and nature of the study, specimen handling and storage. In particular, the information clearly stated the rights of the participants to rigorous privacy measures (non-disclosure clause of personal identifiable data, access by the participant to his/her personal data, right to change or update the personal information provided) and the study's opt-out clause, guaranteeing that every participant may withdraw at any time from the study, for any reason and without consequences. To implement this right, the project has in place several information strategies to keep the communities updated about the development of the research. The study was approved by the Ethics Committee of the Autonomous Province of Bolzano. Every six months the Committee is informed of any changes or developments.

A standardized, interviewer-administered questionnaire was submitted to the participants. The questionnaire was structured as follows: i) reconstruction of family history (pedigree); ii) questions about cardiology, angiology, neurology, psychiatry, ophthalmology, endocrinology, respirology, dermatology, allergy, internal medicine, hematology, oncology, gynecology (for women participants), and injuries due to accidents; iii) life-style exposures (such as smoking habits, alcohol consumption). The typical question occurring in any field of part ii of the questionnaire was a closed question, allowing a yes/no/don't know response. If the interviewing physician had knowledge of whether the subject was affected, this could be indicated, regardless the subject's answer. Subjects were asked the year or age of onset of their condition, they were asked to add a free text description of symptoms (to allow for identification of specific diseases or syndromes) and they were questioned to determine if any relative had the same trait.

Subjects underwent anthropometric measurements (such as height, weight and waist measurements), respiratory and ultrasound-based bone densitometry tests, dioptric measurement of eyeglasses, ECG, and blood pressure measurements. A urine sample was collected from each participant, and blood samples were taken for DNA and serum extraction, and for routine, biochemical analyses, including blood count/differential blood count, Quick, aPTT, ferritine, TSH, T4, cholesterol, HDL, LDL, triglycerides, electrolytes (K, Na, Ca), GPT, GOT, GGT, glucose, and creatinine. A serum sample for each participant was prepared and stored at -80°C for subsequent analysis.

Genomic DNA was isolated from 20 ml of EDTA-treated blood, according to standard procedures [35]. DNA of all 1175 study participants was included in a genome-wide scan performed by deCODE genetics, Iceland, using 1000 microsatellite markers (STRs) evenly spaced across the genome at 4 cM intervals. deCODE's genome scan marker sets are based on the Applied Biosystems HD-5 Linkage Mapping Set and on in-house designed and validated markers originally selected from the Marshfield genetic map. Map order and position were determined from the deCODE Icelandic genetic map [36].

Genealogical information was reconstructed by means of church records (baptisms, marriages, death records, and family books) reaching back to the early 17th century and completed with municipality lists starting in 1924. We started by reconstructing single families, that in a second step were linked together to form genealogical trees of the whole villages. Genealogical data were managed using Cyrillic^© ^2.1 (CyrillicSoftware 2000, Wallingford, UK).

### MICROS-stage 2. Specialist examination and genetic analysis

Subjects are selected on the basis of a reported trait of interest, then they are asked for further examination. As far as possible, the information is extended to the pedigree of the targeted subjects, after all the ethical aspects are taken into account. In case of agreement, targeted patients and relatives are diagnosed by an expert specialist. After collecting the diagnoses, the genetics of the disease is under study, starting with linkage analysis and subsequent association studies.

### Database structure and privacy issues

Data are entered in an enterprise-level database on a web-base front end providing a wide range of tools for entering and querying subject information. The database design was implemented in accordance with the Italian privacy law. Personal identifying data are physically separated from the other data and secured by anonymising methods. A unique code is given to each questionnaire and to the specimens, which are stored in a secure room. A role-based access policy guards access to the database. This system ensures that all information on individuals – genealogical, medical and genetic – is anonymised, identifiable only by authorized persons, who are assigned different levels of access rights to the database according to their role in the research. Transaction logs are maintained to record access to sensitive data. Servers are backed up weekly on tapes and stored off-site in a fire-proof safe.

Furthermore, a java-api for the visual analysis of our complex data was implemented. It provides all the necessary functionality for the interactive exploration and analysis of our extended genealogies [37].

### Statistical analysis

For the purposes of this study, *birthplace *was defined as the village in which the subject's mother was living at the time of the subject's birth. *Educational level *was classified as "no school attended", "primary school" (from 6 to 10 year old), "middle school" (11–14), "vocational school" (usually 15–17), "secondary school" (15–19), and university. Subjects were asked for their *occupations *in the life: a maximum of four jobs could be reported, together with the respective working periods. For the *smoking habits *measure, subjects were defined as "never smokers" (never smoked or smoked for less than 1 year), "past smokers" (stopped smoking more than 1 year before the date of the interview) and "current smokers". *Alcohol consumption frequency *was classified as rarely (never or seldom), often (1 to 4 times per week), daily.

Prevalence and 95% exact confidence intervals (95%CIs) were estimated for the main binary traits. For each trait, the probability of familial clustering (PFC) was assessed through the estimation of a chi-square statistic, which compares the observed with the expected distribution of affected siblings [38,39]. To this aim, we used the GAP software package under R 2.3.1 [40]: due to the high dimensionality of the tables, the enumeration of all possible scenarios was not possible, thus simulated p-values were estimated via Monte Carlo permutations (10 million runs).

Inter-village homogeneity of subject's characteristics and study traits was assessed through chi-square test or F-test, depending on the variable being qualitative or quantitative. Homogeneity of quantitative traits between sex and age classes (18–30, 30–50, 50–70, 70+) was determined using the Kruskal-Wallis test.

Thanks to the collection of extended genealogical trees for the three villages from church and municipal records, and from information on a small number of marriages between two of the three villages, we were able to build a pedigree including participants from all three villages. The accuracy of the pedigree structures was verified by checking available genotype data for Mendelian inconsistencies using Pedstats 0.6.8 [41].

The power of variance component linkage analysis to detect a QTL was assessed via simulation using the SOLAR software package [42]. A biallelic locus was assumed, with minor allele frequency ranging from 0.01 to 0.50; narrow heritability (h^2^) was assumed taking values from 0.10 to 0.60; QTL-specific heritability ranged from 0.05 to h^2^, with 0.05 increments. Since the average inter-marker distance was 4 cM, we assumed the marker be located at 0, 1 or 2 cM, respectively, from the QTL. Simulations were replicated on pedigrees from each single village separately and on the whole pedigree after splitting it with the Greffa software [43]. A LOD-score cutoff of 2 was taken as indication of suggestive linkage, a LOD-score of 3 was taken as a cutoff threshold for significant linkage [44].

## Results

Data were collected from September 2002 to December 2003. A total of 1175 volunteers were enrolled in the study, 509 from Stelvio (percentage of the total population: 48%), 351 from Martello (51%) and 315 from Vallelunga (93%). We obtained screening questionnaire, clinical measurements and genotypic data from each participant.

Complete genealogy was reconstructed for 50,037 individuals, starting from the participants and going back to the 1600s (15 generations). When, for analysis issues, we limited the extension of the pedigree to five generations, 10,630 individuals were included, grouped into 178 pedigrees. One thousand, seven hundred and thirty-two singletons were discarded because it was impossible to include them in any pedigree. One of the 178 pedigrees accounted for 7049 subjects; in the remaining 177 pedigrees, the median number of subjects per pedigree was 8 (range: 3–85). Overall, mean inbreeding coefficient was 0.000565 (standard deviation, sd = 0.004441) and mean kinship coefficient was 0.001777 (sd = 0.014439). When considering village-specific pedigrees, for Vallelunga, Martello, and Stelvio, the mean inbreeding coefficient was 0.000432 (sd = 0.002213), 0.000172 (sd = 0.001367) and 0.000112 (sd = 0.003625), respectively, and the mean kinship coefficient was 0.002228 (sd = 0.014822), 0.002317 (sd = 0.015773), and 0.000806 (sd = 0.012370), respectively.

### Participant's characteristics

**Figure 2:**
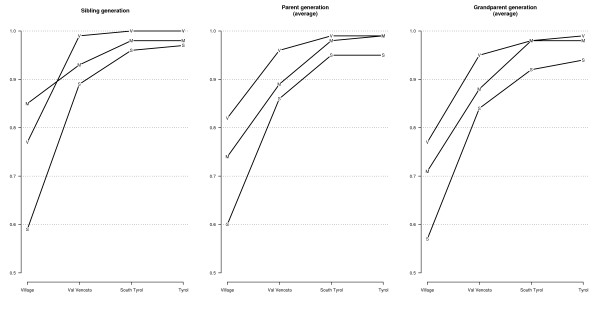
Distribution of participant's birthplaces by village (Stelvio = S, Vallelunga = V, Martello = M) and generation.

The birthplace of participants, their parents and grandparents was recorded: 85% of subjects from Martello, 77% from Vallelunga and 59% from Stelvio were born from a mother who was living in the same village where they were residing at the time of the interview. When extending the area to the Val Venosta, we observed that the percentage of people born in the valley was 89% (Stelvio) to 99% (Vallelunga). A similar pattern was observed for the parent's and grandparent's generations (Figure 2).

Distribution of subject's characteristics is reported in Table 1. Subjects ranged in age 18–88 years (mean = 46.0, sd = 16.3), 56.5% were female. The sex/age distribution was homogeneous between the three villages.

**Table 1:** Distribution of demographic and social characteristics of study participants by village

**Socio-demographic characteristics**		**All villages**		**Stelvio**		**Martello**		**Vallelunga**		p*
		n	%	n	%	n	%	n	%	
	Total	1175	100	509	44.2	351	29.4	315	26.4	
Sex	Males	511	43.5	216	42.5	147	41.9	147	46.8	0.3639
	Females	664	56.5	293	57.5	204	58.1	168	53.2	
Age	Mean, sd	45.9	16.2	46.0	15.7	46.4	15.8	45.1	17.5	0.5907
Education	Elementary school	334	28.4	144	28.2	122	34.7	68	21.7	0.0003
	Middle school	279	23.7	123	24.1	81	23	75	23.9	
	Vocational school	312	26.6	118	23.2	92	26.1	102	32.5	
	Secondary school	205	17.4	96	18.8	47	13.4	62	19.7	
	University	46	3.9	29	5.7	10	2.8	7	2.2	
Occupation	Agriculture	132	11.3	96	18.8	54	15.4	12	3.9	< 0.0001
	Working at home	196	16.7	92	18.2	59	16.9	49	15.6	
	Manual workers	163	13.9	55	10.8	55	15.7	46	14.5	
	Working in a shop	65	5.5	29	5.7	17	4.9	18	5.8	
	Tourism	228	19.4	76	15.0	53	15.1	78	24.9	
	Industry	40	3.4	10	1.9	11	3.1	14	4.4	
	Public employee	72	6.1	26	5.1	21	6.0	21	6.8	
	Teacher	72	6.1	28	5.4	12	3.4	26	8.3	
	Health care personnel	35	3.0	23	4.5	7	2.0	9	2.7	
	Other jobs	110	9.4	39	7.6	42	12.0	27	8.7	
	Not working	64	5.4	36	7.0	19	5.4	14	4.4	
Smoking habits	Never smoker	634	54.0	280	55	188	53.6	166	52.7	0.1635
	Past smoker	265	22.5	107	21.1	92	26.2	66	20.8	
	Current smoker	276	23.4	121	23.8	71	20.2	83	26.5	
Alcohol consumption	Never to seldom	745	63.4	301	59.1	218	62.1	226	71.7	0.0001
	1 to 4 days a week	244	20.8	132	25.9	62	17.7	50	15.9	
	Daily	186	15.9	76	15	71	20.2	39	12.4	

*P-value for the Chi Square test for association between subject's characteristics and village; as long as age was continuous, it was tested with an F-test

Overall, 52.1% of participants went to school until they were 13 years old, 17.4% completed secondary school and 3.9% had a higher degree. The educational level was non homogeneous between villages (p = 0.0003). On average, males had a higher level of education than females (p-value < 0.001) and education was related to age (p-value < 0.001): secondary school or university were attended by 3% of the 70+ year old participants, and by 49% of the 18–30 year old participants.

#### Occupation

Overall, 19.4% were employed in tourism, with noticeable differences between villages (24.9% in Stelvio, 15.0% in Vallelunga). In Vallelunga, the leading sector was agriculture (18.8%). Many people (in particular women) worked at home as housewives. The second most common category of occupation (13.9%) was manual trades, typical of small villages: bricklayers, carpenters, electricians, etc. Ninety-five percent of participants held the same occupation throughout life.

#### Smoking habits

We observed a differential distribution of smoking habits between the two sexes (p < 0.001). Percentage of never smokers was lower for younger males than for older males, but higher for younger females than older females. Overall the smoking prevalence decreased with decreasing age. Smoking habits were homogeneous across villages (p = 0.1635).

#### Alcohol consumption

Eighty-one percent of women and 41% of men reported an alcohol consumption frequency of never to seldom; 27% of males and 7% of females reported daily frequency; males consumed alcohol more often than females (p < 0.001). Alcohol consumption varied by age (p-value < 0.001) and village (p-value = 0.0001).

### Qualitative traits

Distribution of qualitative traits, by village, is reported in Table 2.

**Table 2:** Total and village specific prevalence of selected traits, with 95% Confidence Intervals (95%CI) and Fisher's exact test p-value

**Qualitative Traits**		**All**		**Stelvio**		**Martello**		**Vallelunga**		
		**Prev**	**95%CI**	**Prev**	**95%CI**	**Prev**	**95%CI**	**Prev**	**95%CI**	**p-value**
**Symptoms for cardiovascular disease**	Elevated Blood Pressure	**17.7**	(15.5, 20.0)	**17.5**	(14.3, 21.0)	**24.8**	(20.2, 29.8)	**10.5**	(7.4, 14.4)	**< 0.0001**
	Cardiac Arrhythmias	**19.5**	(17.3, 21.9)	**16.8**	(13.7, 20.2)	**25.2**	(20.7, 30.1)	**17.8**	(13.8, 22.5)	**0.0067**
	Angina pectoris	**16.7**	(14.6, 19.0)	**11.9**	(9.2, 14.9)	**26.5**	(22.0, 31.4)	**13.9**	(10.3, 18.2)	**< 0.0001**
	Cardiac Disease	**2.5**	(1.7, 3.6)	**1.9**	(0.9, 3.5)	**3.7**	(2.0, 6.3)	**2.2**	(0.9, 4.5)	0.2476
	Myocardial Infarction	**1.2**	(0.6, 2.0)	**1.3**	(0.5, 2.7)	**1.4**	(0.5, 3.3)	**0.6**	(0.1, 2.3)	0.6176
	Heart Defects	**3.2**	(2.3, 4.4)	**2.8**	(1.6, 4.6)	**4.0**	(2.2, 6.6)	**2.9**	(1.3, 5.4)	0.5882

**Neurological traits**	Vertigo	**32.0**	(29.3, 34.7)	**34.4**	(30.3, 38.6)	**27.5**	(22.8, 32.5)	**33.0**	(27.9, 38.5)	0.0900
	Migraine	**20.8**	(18.6, 23.2)	**20.5**	(17.1, 24.2)	**25.1**	(20.7, 30.0)	**16.6**	(12.6, 21.1)	**0.0244**
	Carpal Tunnel Syndrome symptoms	**14.8**	(12.7, 17.1)	**20.2**	(16.2, 24.6)	**12.0**	(8.8, 15.9)	**11.5**	(8.2, 15.5)	**0.0017**
	Restless Leg's Syndrome (RLS)	**12.8**	(10.9, 14.8)	**13.3**	(10.5, 16.6)	**12.6**	(9.3, 16.6)	**12.0**	(8.7, 16.1)	0.8729
	Vascular Problems in the Carotid Arteries	**2.9**	(2.0, 4.0)	**2.3**	(1.2, 4.0)	**3.5**	(1.8, 6.0)	**3.2**	(1.5, 5.8)	0.5677
	Stroke	**1.7**	(1.0, 2.6)	**1.2**	(0.4, 2.5)	**2.6**	(1.2, 4.8)	**1.6**	(0.5, 3.7)	0.2714
	Epilepsy	**2.0**	(1.3, 3.0)	**2.7**	(1.5, 4.4)	**2.0**	(0.8, 4.1)	**1.0**	(0.2, 2.8)	0.2337

**Symptoms related to the respiratory system, allergy and dermatology**	Asthma	**4.2**	(3.1, 5.5)	**4.8**	(3.1, 7.0)	**4.3**	(2.4, 7.0)	**3.2**	(1.5, 5.8)	0.5566
	Chronic Bronchitis	**5.2**	(4.0, 6.6)	**5.1**	(3.4, 7.4)	**6.9**	(4.4, 10.0)	**3.5**	(1.8, 6.2)	0.1519
	Hay Fever	**8.3**	(6.8, 10.0)	**9.4**	(7.0, 12.2)	**6.3**	(4.0, 9.4)	**8.6**	(5.7, 12.3)	0.2586
	Insect Hypersensitivity	**12.7**	(10.9, 14.7)	**12.6**	(9.9, 15.7)	**12.4**	(9.1, 16.3)	**13.3**	(9.8, 17.6)	0.9252
	Eczema	**3.9**	(2.9, 5.1)	**5.3**	(3.6, 7.6)	**3.5**	(1.8, 6.0)	**1.9**	(0.7, 4.1)	**0.0403**
	Psoriasis	**3.2**	(2.3, 4.4)	**3.6**	(2.2, 5.6)	**2.0**	(0.8, 4.1)	**3.8**	(2.0, 6.5)	0.3134

**Other traits**	Diabetes	**3.6**	(2.6, 4.9)	**3.5**	(2.1, 5.4)	**4.9**	(2.9, 7.7)	**2.5**	(1.1, 4.9)	0.2680
	Goitre	**6.4**	(5.1, 7.9)	**5.9**	(4.0, 8.3)	**7.7**	(5.2, 11.0)	**5.7**	(3.4, 8.9)	0.4865
	Gout or Hyperuricemia	**3.5**	(2.5, 4.7)	**3.6**	(2.2, 5.6)	**4.6**	(2.6, 7.3)	**1.9**	(0.7, 4.1)	0.1470
	Gallbladder Disease	**10.7**	(9.0, 12.9)	**12.2**	(9.5, 15.3)	**10.9**	(7.8, 14.6)	**7.9**	(5.2, 11.5)	0.1454
	Ulcer	**9.3**	(7.7, 11.1)	**10.7**	(8.2, 13.7)	**8.0**	(5.4, 11.4)	**8.3**	(5.5, 11.9)	0.3280
	Liver Disease	**6.3**	(5.0, 7.9)	**6.1**	(4.2, 8.6)	**9.2**	(6.4, 12.8)	**3.5**	(1.8, 6.2)	**0.0102**
	Kidney Disease	**13.6**	(11.7, 15.7)	**13.1**	(10.3, 16.3)	**14.7**	(11.1, 18.8)	**13.3**	(9.8, 17.6)	0.7877
	Anemia	**6.5**	(5.1, 8.0)	**5.8**	(3.9, 8.2)	**8.5**	(5.8, 12.0)	**5.4**	(3.2, 8.5)	0.2038

#### Symptoms for cardiovascular disease (CVD)

Elevated blood pressure, cardiac arrhythmias, and angina pectoris were the most common disorders; all the three with a non-homogeneous distribution across villages. Prevalence of these symptoms steeply increased with age. The percentage of women reporting symptoms was higher than males, but this difference varied with age. Cardiac disease was reported by 2.5% (95%CI: 1.7–3.6%) of subjects, with 1.2% (95%CI: 0.6–2.0%) having had a myocardial infarction. Interestingly, 36 subjects reported heart defects, such as heart failure and coronary heart disease (3.2%, 95%CI: 2.3–4.4%). The last three symptoms were homogeneously distributed across villages. For all the CVD symptoms, Martello had the highest prevalence. For sex-age specific distribution of the main CVD symptoms [see additional file 1].

#### Neurological traits

The most frequent neurological symptoms were vertigo and migraine. Migraine prevalence was higher in Martello and Vallelunga than in Stelvio (p = 0.0244). Symptoms compatible with carpal tunnel syndrome were reported by 14.8% (95%CI: 12.7–17.1%), with Vallelunga having much higher prevalence than the other two villages (p = 0.0017). Prevalence of restless leg's syndrome (RLS) was 12.8% (95%CI: 10.9–14.8%). Three percent (95%CI: 2.0–4.0%) had vascular problems in the carotid arteries, 1.7% had a stroke (95%CI: 1.0–2.6%) and 2.0% (95%CI: 1.3–3.0%) suffered from epilepsy.

#### Symptoms related to the respiratory system, allergy and dermatology

Any episode of asthma in life was reported by 4.2% (95%CI: 3.1–5.5%) of subjects; 5.2% (95%CI: 4.0–6.6%) reported to have had chronic bronchitis and 8.3% (95%CI: 6.8–10.0%) reported hay fever. At younger ages asthma prevalence was higher in women than in men, with the percentage increasing with age until more males than females were affected by asthma after the age of 70. A similar pattern of increasing prevalence with age was observed for chronic bronchitis, but with females always having a higher prevalence than males. An almost parallel decreasing trend was observed for hay fever [see additional file 1]. Hypersensitivity to any insect was quite common (12.7%, 95%CI: 10.9–14.7%). Four percent (95%CI: 2.9–5.1%) reported eczema and 3.2% (95%CI: 2.3–4.4%) psoriasis. Eczema prevalence was lower in Martello than in other villages (p = 0.0403).

#### Other symptoms

Diabetes prevalence was 3.6% (95%CI: 2.6–4.9%). Goitre was present in 6.4% (5.1–7.9%) and gout or hyperuricemia in 3.5% (95%CI: 2.5–4.7%) of the sample. Eleven percent (95%CI: 9.0–12.6%) reported gallbladder disease, 9.3% (95%CI: 7.7–11.1%) had ulcer and 6.3% (95%CI:5.0–7.9%) had liver disease. Between village heterogeneity of liver disease was observed (p = 0.0102). Kidney disease was reported by 13.6% (95%CI: 11.7–15.7%) and anemia by 6.5% (95%CI: 5.1–8.0%).

#### Probability of familial clustering

The hypothesis of random distribution of a trait across the pedigree was rejected for all the traits related to CVD: the p-values ranged from < 0.000001 for elevated blood pressure to 0.0295 for cardiac arrhythmias. When assessing the likelihood of familial clustering within village, certain heterogeneity was observed. Elevated blood pressure clustered in families in each of the three villages, but cardiac arrhythmias was significantly clustered in families only in Martello, and myocardial infarction as well. Familial clusters of myocardial infarction and heart defects were observed in Stelvio and Vallelunga, but not in Martello. Clusters of angina pectoris were assessed in Stelvio. Among the neurological traits, vascular problems at the level of carotid arteries had the strongest evidence of familial clustering (p < 0.000001). Other traits clustering in families were stroke, apoplexy, migraine, Alzheimer's disease, CTS-like symptoms, and RLS. Similar to the case of CVDs, some inter-village differences were observed. While some symptoms were clustering in families in all of the three villages (CTS, RLS, and migraine), in other cases this evidence was related to one single village (e.g.: stroke). Asthma, chronic bronchitis, and diabetes showed evidence of familial clustering in Stelvio and Martello. Ulcer and liver disease were clustering in families in Martello and Vallelunga. No evidence of familial clustering was observed for kidney and gallbladder diseases. More results are reported in Table 3.

**Table 3:** Probability of Familial Clustering: Monte Carlo simulated p-values for some selected traits in the three microisolates separately and on the pooled data

**Traits**	**Stelvio**	**Martello**	**Vallelunga**	**Pooled data**
**Symptoms for cardiovascular disease**				
Elevated Blood Pressure	**0.0157**	**0.0035**	**0.0006**	**< 0.0001**
Cardiac Arrhythmias	0.0651	**0.0213**	0.5690	**0.0295**
Angina pectoris	**0.0005**	0.6729	0.2500	**0.0061**
Cardiac Disease	**0.0026**	0.2319	**0.0138**	**0.0000**
Myocardial Infarction	0.6235	**0.0003**	0.2571	**0.0000**
Heart Defects	**0.0016**	0.6294	**0.0279**	**0.0048**
				
**Neurological traits**				

Vertigo	**0.0258**	0.1831	0.2167	0.1750
Migraine	**< 0.0001**	0.3916	**0.0005**	**< 0.0001**
Carpal Tunnel Syndrome symptoms	**0.0027**	**0.0303**	**0.0000**	**< 0.0001**
Restless Leg's Syndrome (RLS)	**0.0109**	0.0646	**0.0008**	**< 0.0001**
Vascular Problems in the Carotid Arteries	**0.0027**	**0.0006**	**0.0025**	**< 0.0001**
Stroke	0.3660	**0.0180**	0.2482	**0.0059**
Epilepsy	**0.0427**	0.7504	0.8637	0.1561
				
**Symptoms related to the respiratory system, allergy and dermatology**				

Asthma	**0.0001**	**0.0005**	0.6379	**< 0.0001**
Chronic Bronchitis	**0.0133**	**0.0396**	0.4507	**0.0007**
Hay Fever	0.1542	**0.0207**	0.1592	0.0916
Insect Hypersensitivity	0.0863	**0.0237**	0.2724	0.0620
Eczema	0.4639	**0.0138**	**0.0012**	**0.0011**
Psoriasis	**0.0118**	1.0000	0.3600	**0.0148**
				
**Other traits**				

Diabetes	**0.0001**	**0.0003**	0.5314	**< 0.0001**
Goitre	0.0605	0.1413	**0.0420**	0.0639
Gout or Hyperuricemia	0.7653	**0.0053**	1.0000	**0.0184**
Gallbladder Disease	0.2856	0.4940	0.0535	0.3141
Ulcer	0.0616	**0.0010**	**0.0001**	**< 0.0001**
Liver Disease	0.6466	**0.0050**	**0.0144**	**0.0163**
Kidney Disease	0.9723	0.3993	0.2123	0.4940
Anemia	0.1978	**0.0211**	0.2789	**0.0070**

### Quantitative traits

Body Mass Index (BMI) differed between sexes and increased with age (minimum median = 21 in 18–30 year old women, maximum median = 28 in 70+ year old women). Waist circumference was related to age, with young males having bigger waist circumference than young females. Among the other determinants for CVD, diastolic and systolic blood pressure increased with age (younger males had higher blood pressure than young females, p < 0.00001). Cholesterol increased with age; in the elderly, females had slightly higher values than males. Mean High Density Lipoprotein (HDL) was 53 mg/dl (sd = 13) in males, 64 mg/dl (sd = 14) in females. Low Density Lipoproteins (LDL) increased with age: mean = 104 mg/dl (sd = 32) in 18–30 year old subjects, mean = 136 (sd = 31) in 70+ year old subjects. Triglycerides were heterogeneous between groups (sex and age, p-value < 0.000001), with median within 75–120 mg/dl. For distribution of CVD traits by age and sex [see additional file 2].

Within the CVD/kidney related traits, median blood sodium level was 138–139 mEq/l and median potassium level was in 4.2–4.3 mEq/l. Higher calcium levels were observed in younger subjects and in males. Hemoglobin was higher in males than in females. Mean creatinine was 0.98 mg/dl (sd = 0.15) in males and 0.79 mg/dl (sd = 0.15) in females. Regarding the intermediate phenotypes for renal function, the distribution of albuminuria was similar across sex-age groups (median within 6–7 mg/l, quartile range: 5–15), with a peak in 70+ year old males, who had the highest values (median = 11 mg/l, quartile range: 8–23). Creatinuria was lower for the elderly and for females: maximum median was 188 mg/dl in 18–30 year old males; minimum median was 74 mg/dl in 70+ year old females.

Fasting glucose was sex-age related, with median within 77–87 mg/dl; among 50+ year old subjects the variability increased as well as hyperglycemia cases. Liver transaminases (glutamic-oxaloacetic transaminase, GOT; glutamate pyruvate transaminase, GPT) and gamma glutamyl-transpeptidase (GGT) had similar patterns: before the age of 70, they were higher in males than in females, after which levels converged to similar values [see additional file 3].

Among anemia related traits, ferritin was higher for males than females: in males the median ranged in 109–142 ng/ml, with no age trend; in females the values increased from a median of 22 in 18–30 year old subjects to 76 in the 70+ year old participants (the variability increasing as well). Mean corpuscular hemoglobin concentration (MCHC) decreased slightly with age (mean = 35 g/dl, sd = 0.8). Red cell distribution width (RDW) was constant until 50 year of age (median 12.3–12.4%), then increased (median ≥ 12.90% after 70 years). Mean corpuscular volume (MCV) was homogeneous between groups (mean = 88 fl, sd = 5).

Immune system indicators exhibited little heterogeneity. Neutrophils were homogeneous between sexes, with small variations and no age trend (median = 48.0–54.2%, with a peak in the 50–70 age class). Lymphocytes were decreasing with age (women: median from 39.5 to 34.1%; men: 38.1 to 31.7%), with females having higher values than males. Heterogeneity (p = 0.00156) but no age trend was observed for monocytes, with males having always higher values than females (p < 0.00001). Median percentage of eosinophils was within 2.3–3.1, with significant sex-age heterogeneity. Basophils had median of 0.7% (interquartile range: 0.5–1.0).

Statistically significant, between-village heterogeneity was observed for all the studied traits but waist circumference, GOT, and several hematological parameters (WBC, HGB, MCV, RDW, PLT, MPV, PCT, and PDW).

### Power of the pedigree for detecting linkage

We ran power simulation for the pooled pedigree (the three villages together), for each one of the three villages separately, and for the total pedigree after pedigree splitting.

**Figure 3:**
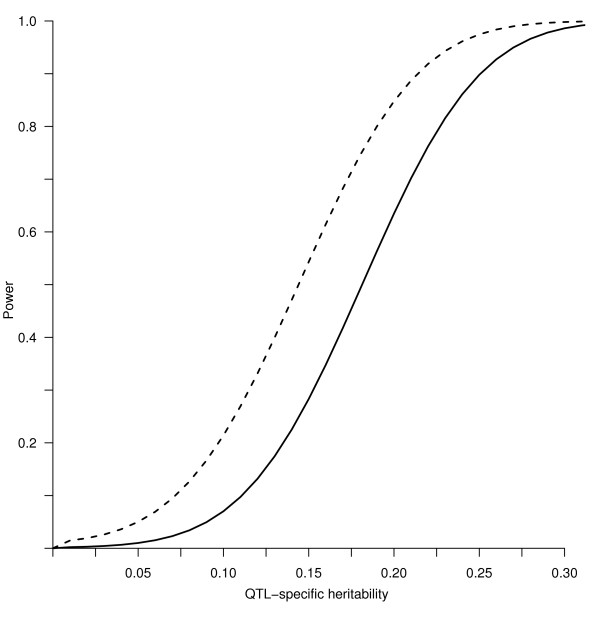
Power of the pooled pedigree to detect a LOD score ≥ 2 (dashed line) and ≥ 3 (solid line).

When considering the pooled data, the power for localizing a QTL was substantial (Figure 3). When the narrow heritability was above 30%, for a QTL-specific heritability of around 18%, the probability to find a suggestive LOD-score (that is, LOD ≥ 2) was 80%. The pedigree had 80% of power to find a LOD-score ≥ 3, the threshold for detecting linkage, for QTLs with 22% of specific heritability.

By pooling all the samples together, we were making the assumption that people from the three microisolates share the same variants with the same frequencies and, consequently, the same effects. In the case that the three microisolates are genetically differentiated, this assumption could be unrealistic. In addition, running a linkage analysis on pedigrees with size 7049 could be unfeasible for computational power limitations. For these reasons we ran more simulations for each microisolate separately.

**Figure 4:**
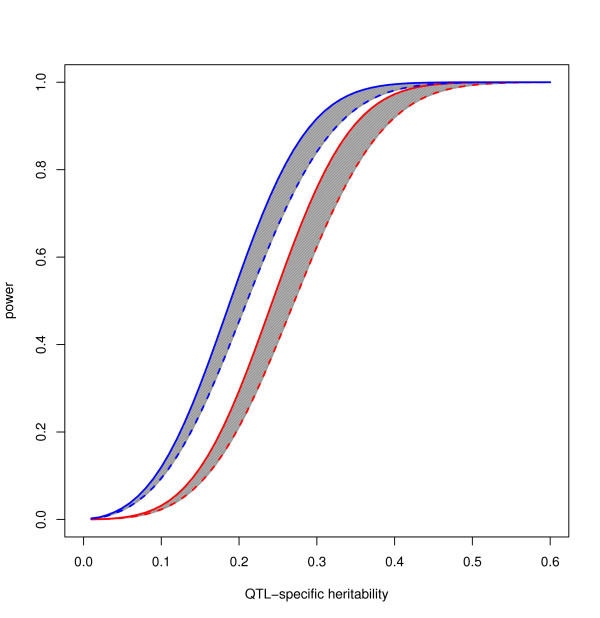
Power of the Vallelunga pedigree to detect a LOD score ≥ 2 (blue line) and ≥ 3 (red line) when the marker is placed 0 (solid line) or 2 (dashed line) cM apart from the QTL. The grey area indicates the intermediate power for markers placed between 0 and 2 cM from the QTL. Narrow heritability = 0.60.

We obtained the best results when analyzing the Vallelunga pedigree, probably because the collection of the 93% of the total inhabitants allowed a better pedigree reconstruction. In Figure 4 we report the power curve for the Vallelunga pedigree when h^2 ^= 0.60. Red and blue lines indicate the probability to find a LOD-score ≥ 3 and ≥ 2, respectively. Power curves for markers placed 0 (continuous line) and 2 (shaped line) cM apart from the QTL are plotted. The grey area within the curves indicates the differential power at different marker-QTL distances. As long as the average microsatellite spacing is 4 cM, it is worth noting that placing the marker at 2 cM from the QTL corresponds to the worst scenario, while 0 cM corresponds to the best one. Depending on location, we observed 80% probability to find a LOD-score ≥ 3 for QTLs with specific heritability from 0.31 (0 cM) to 0.35 (2 cM). When the QTL specific heritability is 25 (0 cM) to 28 (2 cM), the Vallelunga pedigree has 80% power to find a suggestive linkage signal (LOD-score ≥ 2).

When simulating power for QTLs with different minor allele frequencies (MAF), we did not observe any particular relationship between MAF and power.

Finally, we considered the option to analyze the pooled dataset after splitting the big pedigrees into smaller ones. Under the constraints of pedigree size ≥ 3 and kinship coefficient ≥ 0.0000125, we obtained the entire sample be split into 78 pedigrees, with 4 to 397 subjects each (mean = 29, median = 13). The average mean kinship coefficient was 0.13 (min = 0.02, max = 0.25). This approach had the advantage to consider all the three villages together while enormously reducing the computational time. Unfortunately, despite these major advantages, the estimated power was lower than considering the Vallelunga microisolate alone, for all the described scenarios.

## Discussion

MICROS is an extensive, population-based survey on three German-speaking microisolates of South Tyrol, with the goal of genetic characterization of the most relevant traits for this population. In this paper we described the first stage of the survey, the study design and the data collection. As described in the methods section, a second stage is necessary to study the traits in a greater detail and, possibly, to recruit subjects that did not participate in the first phase, so that families could be completed. The second assessment phase requires contacting the GPs and the targeted families, in order to narrowly define specific phenotypes. To narrow the phenotype definition, subjects are then re-examined by specialists for the medical fields of interest.

Among the strengths of the MICROS study, the interdisciplinary scientific team is one of the most important. The interconnection between historians and biologists-geneticists was the first step toward the targeting of the microisolates. Moreover, geneticists, ethicists and epidemiologists had a key role in the design of the survey. The correct data processing is being guaranteed by the bioinformatics core. At the same time, research is ongoing on all of these fields.

After demonstrating the historical isolation of South Tyrol across the centuries, we concluded that this isolation still plays an important role in the microisolates; the analysis of demographical records highlighted that the population is very stable and the absolute number of inhabitants almost the same across the years [22]. We demonstrated that participants were used to live in the same place where they were born (Figure 2). Between 90 and 100% of the subjects were born within the Val Venosta, making this aggregate like a closed community. This custom was confirmed also for the parental and grandparental generations. The preference for the birthplace had, in our population, higher rates than in other very isolated populations such as those living in Sardinia [45]. The high percentage of endogamy and of consanguineous unions observed in these villages [33] could be a consequence of this temporal closeness. The temporal stability was also reflected in the occupational status of the participants: 95% never changed jobs within their lifetime. People moved very rarely from home and then only for short periods. In fact, the number of people with an university degree was negligible, perhaps also because the closest university was over 100 km far away.

All these features lead us to consider the possibility of upper Val Venosta itself as a secondary isolate [34] with several isolated units (which we termed microisolates) characterised by an even higher extent of linkage disequilibrium [20].

We have listed a number of traits, classifying qualitative symptoms by those categories which will be the most relevant for the involved clinicians. Interestingly, many common traits showed a significant probability of familial clustering. On the one hand, we observed some traits clustering in families within all three villages (e.g.: elevated blood pressure and some neurological traits); on the other hand, familial clusters were observed only in one or two villages as, for example, the case of angina pectoris, migraine, asthma, and liver disease. Familial clustering can be driven by allelic homogeneity or common life style. The combination of these two factors must be carefully evaluated for each single trait under study and simplyfing assumption on the allelic distribution cannot be done.

Until now, we have not performed further investigations on the qualitative traits obtained through the screening questionnaire, because these traits are intended to serve as indicators of the possible presence of specific phenotypes, which will be investigated in the second stage of the study. At that stage, clinical diagnoses will be performed, and pedigrees will be reconstructed starting from affected subjects. This was the case for RLS which was assessed in a greater detail with clinical diagnosis by a movement disorder specialist and successive linkage analysis [21]. A genome-wide linkage scan was carried out, which led to the discovery of a novel locus on chromosome 2q [16], thus suggesting the potential in our pedigree for detecting qualitative trait loci.

Regarding the power to detect QTLs, the MICROS study has a potentially high power when pooling data from the three villages. Unfortunately, this potentially big power is bounded by the limited computational power of linkage analysis softwares. Nevertheless, the power resulting from the Vallelunga pedigree of finding LOD-scores ≥ 2 is encouraging and suggests that the villages of Martello and Stelvio could be considered as replication samples for common variants. The results also suggest that some efforts should be spent in second phase studies for phenotyping and genotyping new individuals, related to the current participants, especially from Martello and Stelvio. Currently, this strategy is being pursued.

The collected sample will also serve for association studies, while controlling for disease specific determinant and effect confounders. We expect that the assessment of phenotype-genotype association could be favoured by the high extent of linkage disequilibriumobserved in the villages [20]. Methods taking into account the non independence of observations are now available, such as family based association methods [46] and general linear and non linear mixed effect models. So, especially for quantitative traits, genowide association scans would be a valid tool to detect causal alleles.

In the case of Vallelunga, where the sample was 93% of all the inhabitants, data will allow researchers to assess the global health of population living in the village, with interesting issues for public health. The observed heterogeneity in the distribution of several qualitative and quantitative traits suggests that, for both linkage and association studies, covariates such as age, sex and environmental factors should be taken into account. Adjustment or stratification may be required, depending on the specific situation.

Within the neurological diseases, further assessment on the RLS is ongoing. In addition, as one out of five participants was affected by migraine, a new research branch on this disease is to be started. At the moment, a questionnaire for the assessment of migraine is under validation. In case of positive response, it will be used to assess migraine in the study subjects. Subjects reporting migraine have been targeted together with relatives which did not participate in the first phase. Following this, a comprehensive study of the genetic epidemiology of this disease and genome-wide linkage analyses will be undertaken.

Genome-wide scans will allow detailed investigation of nephrological diseases, and of gall bladder disease, in particular gall stone formation. Chronic kidney disease (CKD) will be studied by means of a quantitative trait, the glomerular filtration rate (GFR), obtained from serum and urine creatinine measurements. Epidemiology of CKD will be explored particularly in the isolated village of Vallelunga, where we have a sample which is representative of the population. Moreover, linkage analysis will be performed together with an association study with environmental and genetic factors.

## Conclusion

Our approach, involving different scientific disciplines, seems an advantageous strategy to define and study small population isolates. Priming of the population and support by the local GPs is a prerequisite to ensure a high participation rate. The isolation of the Alpine populations that we included in our study together with the extensive data collection make the MICROS study a powerful resource for the study of diseases in many fields of medicine. It is a promising strategy to ascertain extended pedigrees ensuring a homogeneous environment. Linkage disequilibrium and historical data from the South Tyrol region, and the small isolated villages suggest that individuals may share a small number of disease alleles. Recent successes and simulation studies give us confidence that our pedigrees can be valuable both in finding new candidates loci and to confirm existing candidate genes.

## Competing interests

The author(s) declare that they have no competing interests.

## Authors' contributions

All authors contributed to drafting the manuscript. The main text was written by PC, MF, and RA with comments and amendments made by all authors. PPP was the study coordinator. PPP, MT, VFD, PGK, and PI conceived of the study and participated in its design. DGA and BVC participated in the study design. PC and MF did the statistical analyses and power simulations. RA, DCU, GM and PGK collected the genealogical data and helped in the draft of the historical paragraphs. MD, EC and FC wrote all the parts related to ethics, privacy issues and database administration. SSA designed the database and the data entry tool, and was involved in the study design. DGA, BVC, PI and PS carried out the DNA extraction, quality controls, sample storage management, and the molecular genetic studies. EA was responsible for data collection and database quality control. Clinical data were commented by WCJ and PPP. All authors have read and approved the final manuscript.

## Pre-publication history

The pre-publication history for this paper can be accessed here:



## Supplementary Material

Additional file 1 Distribution of cardiovascular and respiratory traits. Prevalence of the main cardiovascular disease symptoms (upper panel) and respiratory traits (lower panel), by age and sex (solid line = females, dashed line = males), with 95% confidence intervals. Click here for file

Additional file 2 Distribution of cardiovascular disease's determinants. Differential distribution of six quantitative traits by age and sex (males: gray; females: white). Age is reported on the x-axis. Click here for file

Additional file 3 Distribution of liver-related intermediate phenotypes. Distribution of glutamic-oxaloacetic transaminase (GOT), glutamate pyruvate transaminase (GPT) and gamma glutamyl-transpeptidase (GGT), by age and sex (gray = males, white = females). Click here for file
