# Supplementary figures and images for: The genetic study of three population microisolates in South Tyrol (MICROS): study design and epidemiological perspectives
Source: BMC Med Genet. 2007 Jun 5;8:29. doi: 10.1186/1471-2350-8-29 (PMC1913911; doi:10.1186/1471-2350-8-29)

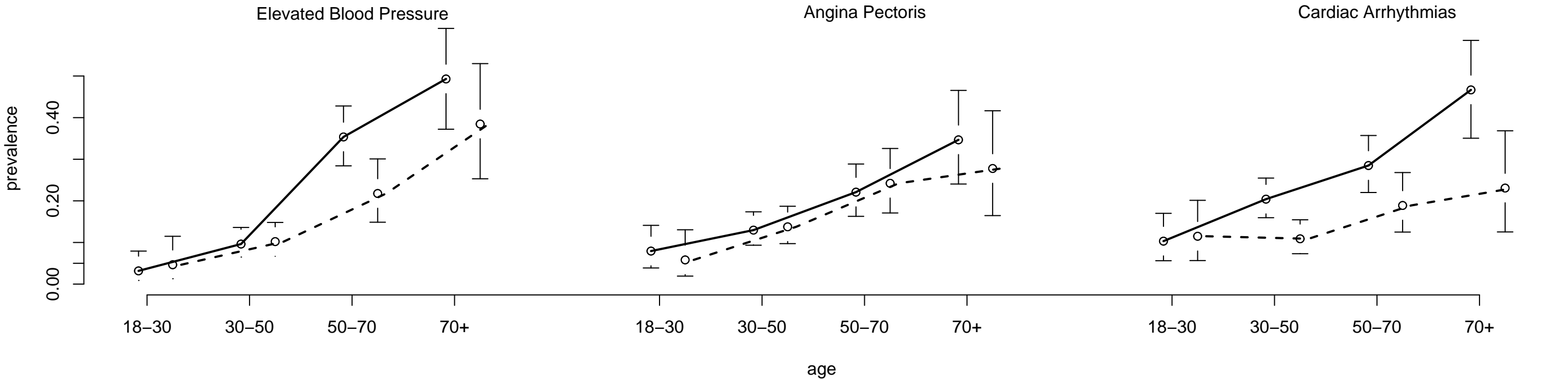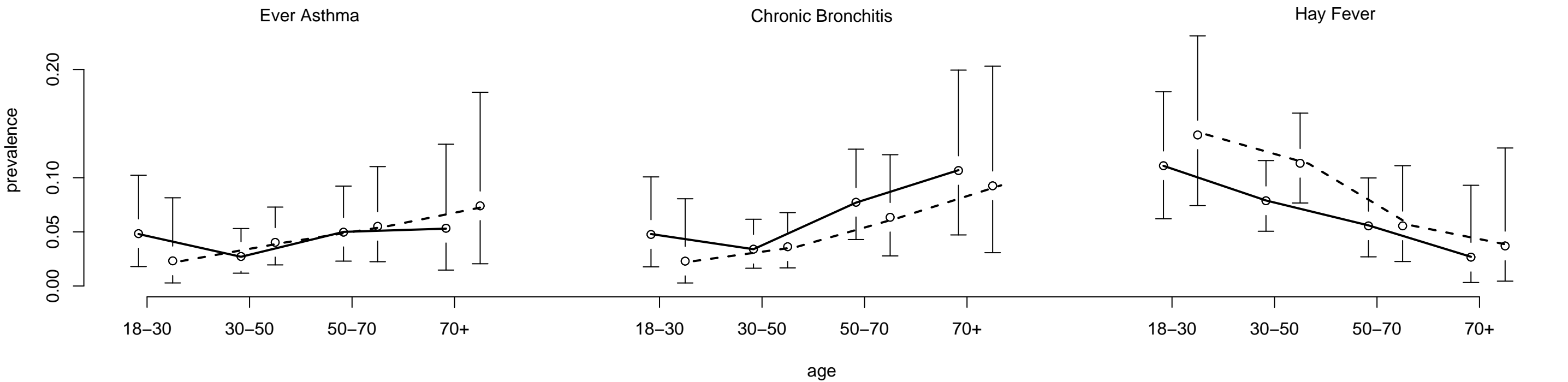

Supplement: Additional file 1 — Distribution of cardiovascular and respiratory traits. Prevalence of the main cardiovascular disease symptoms (upper panel) and respiratory traits (lower panel), by age and sex (solid line = females, dashed line = males), with 95% confidence intervals. [file 1471-2350-8-29-S1.pdf]

**BMI**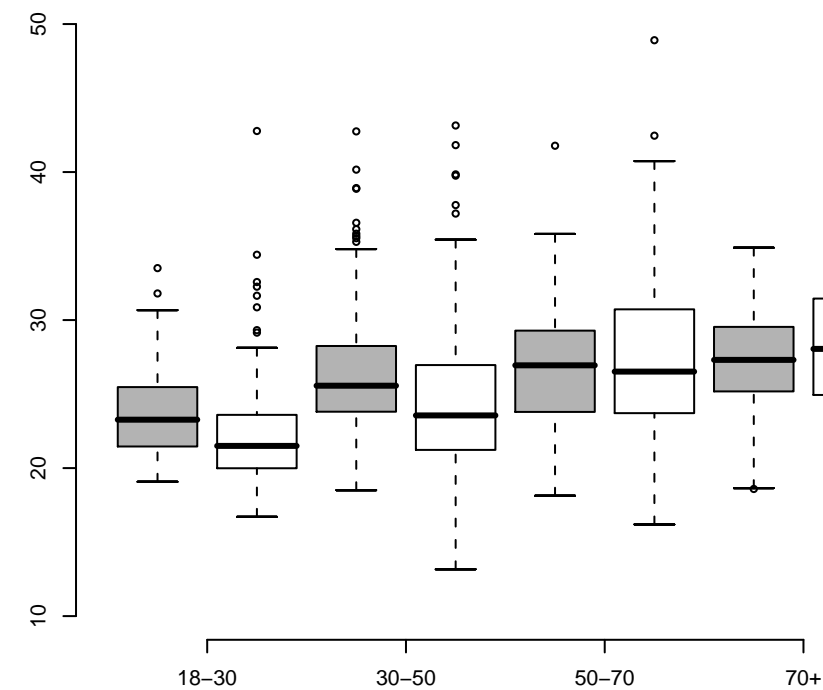**Waist (cm)**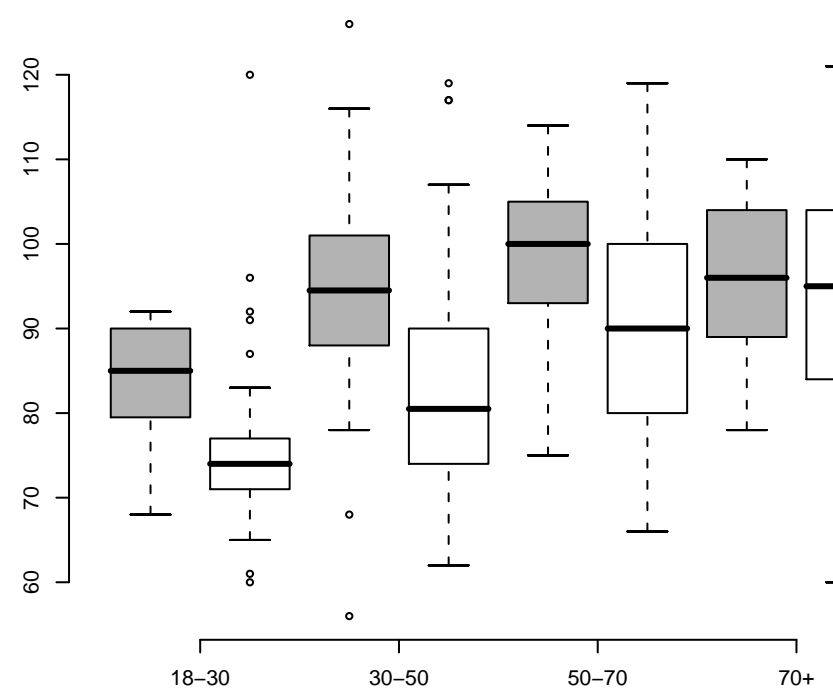**Cholesterol (mg/dl)**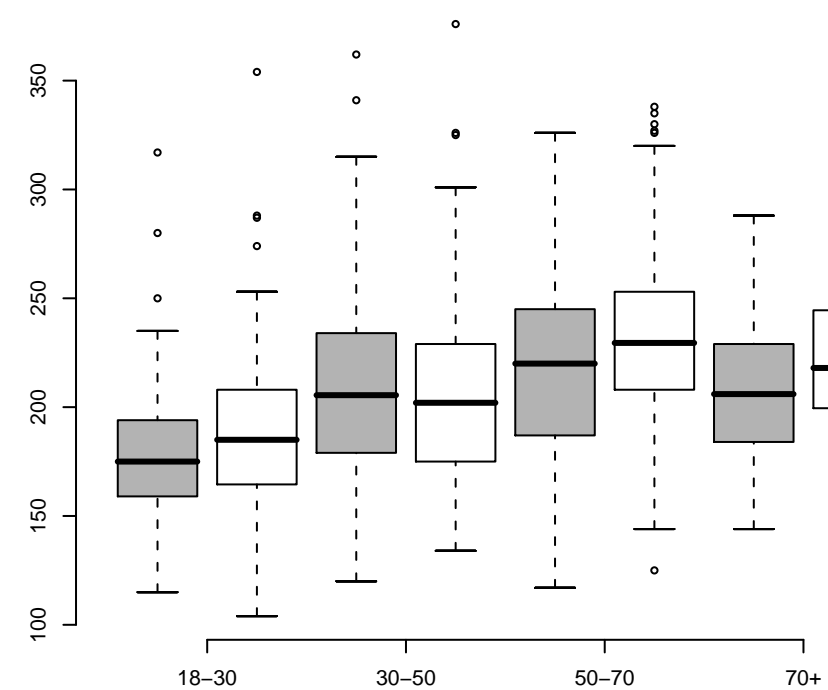**Hemoglobin (g/dl)**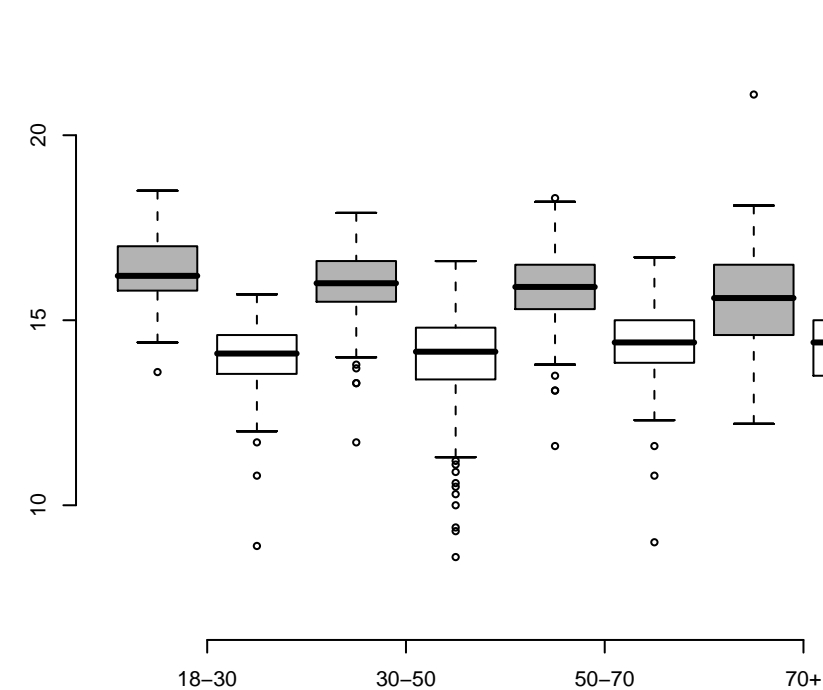**Ferritin (ng/ml)**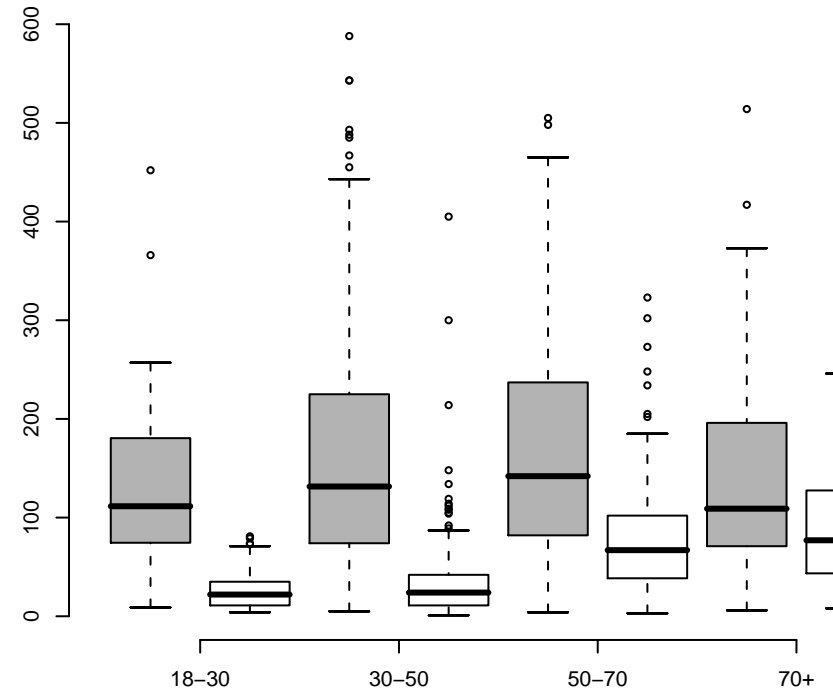**Glucose (mg/dl)**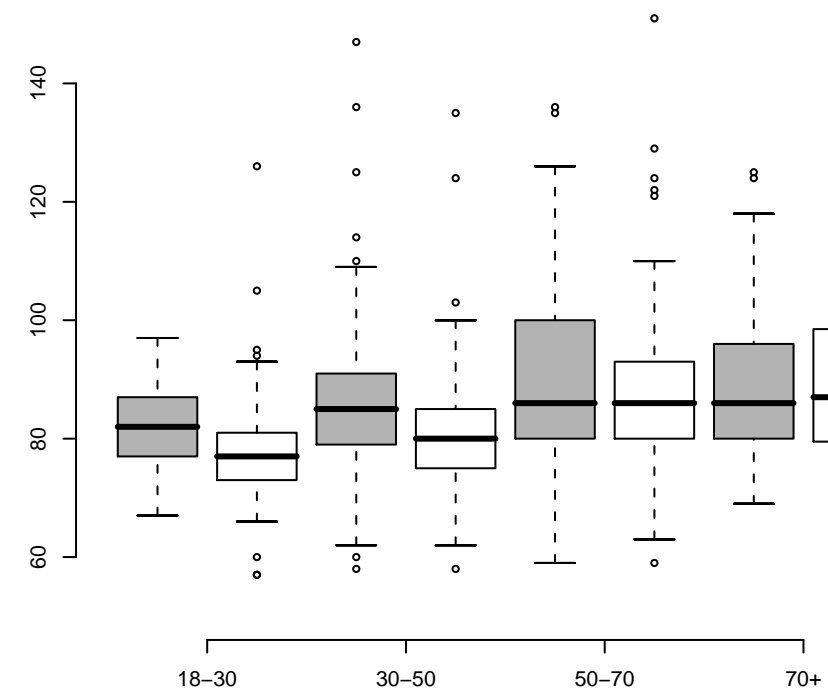

Supplement: Additional file 2 — Distribution of cardiovascular disease's determinants. Differential distribution of six quantitative traits by age and sex (males: gray; females: white). Age is reported on the x-axis. [file 1471-2350-8-29-S2.pdf]

GOT (U/l)

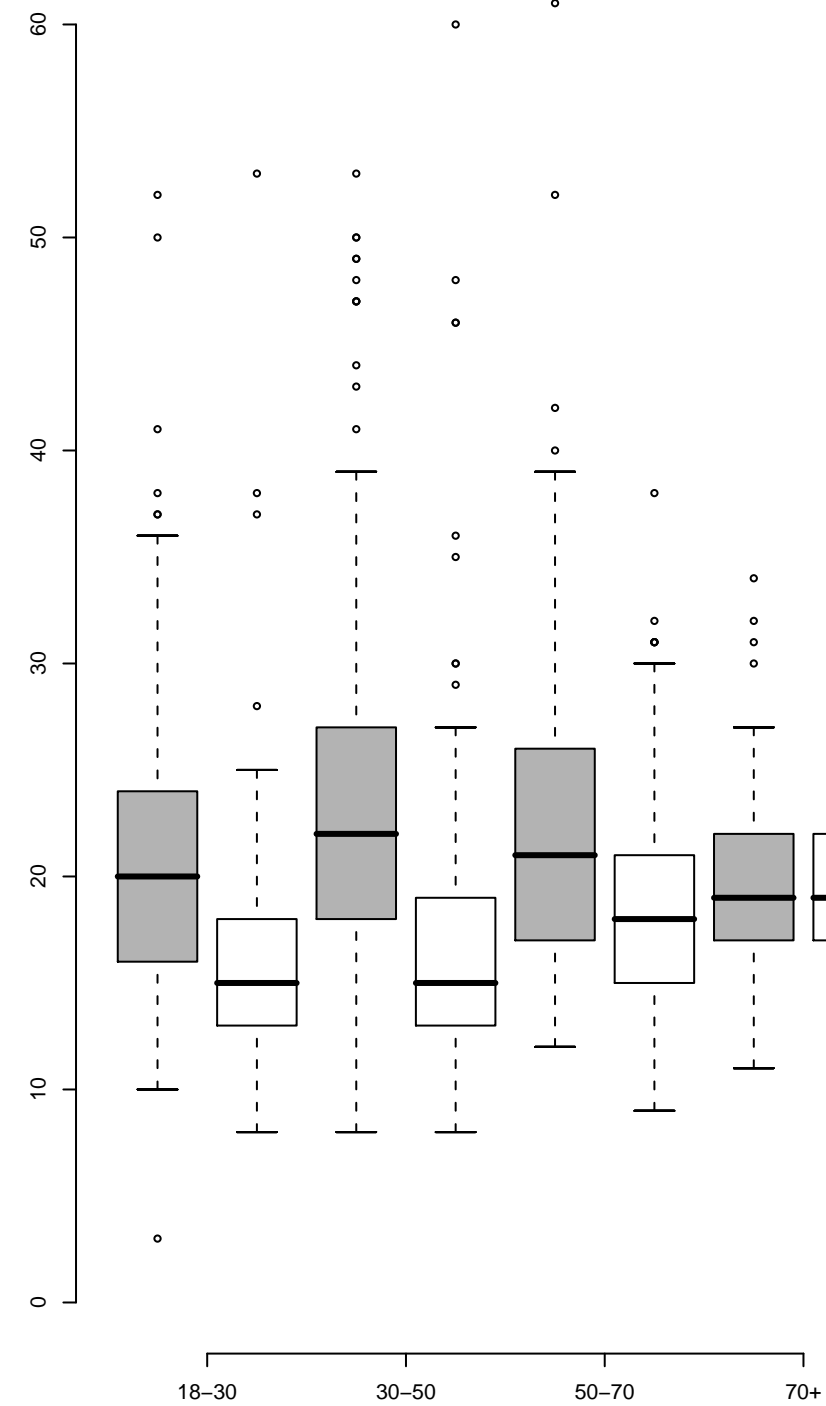

GPT (U/l)

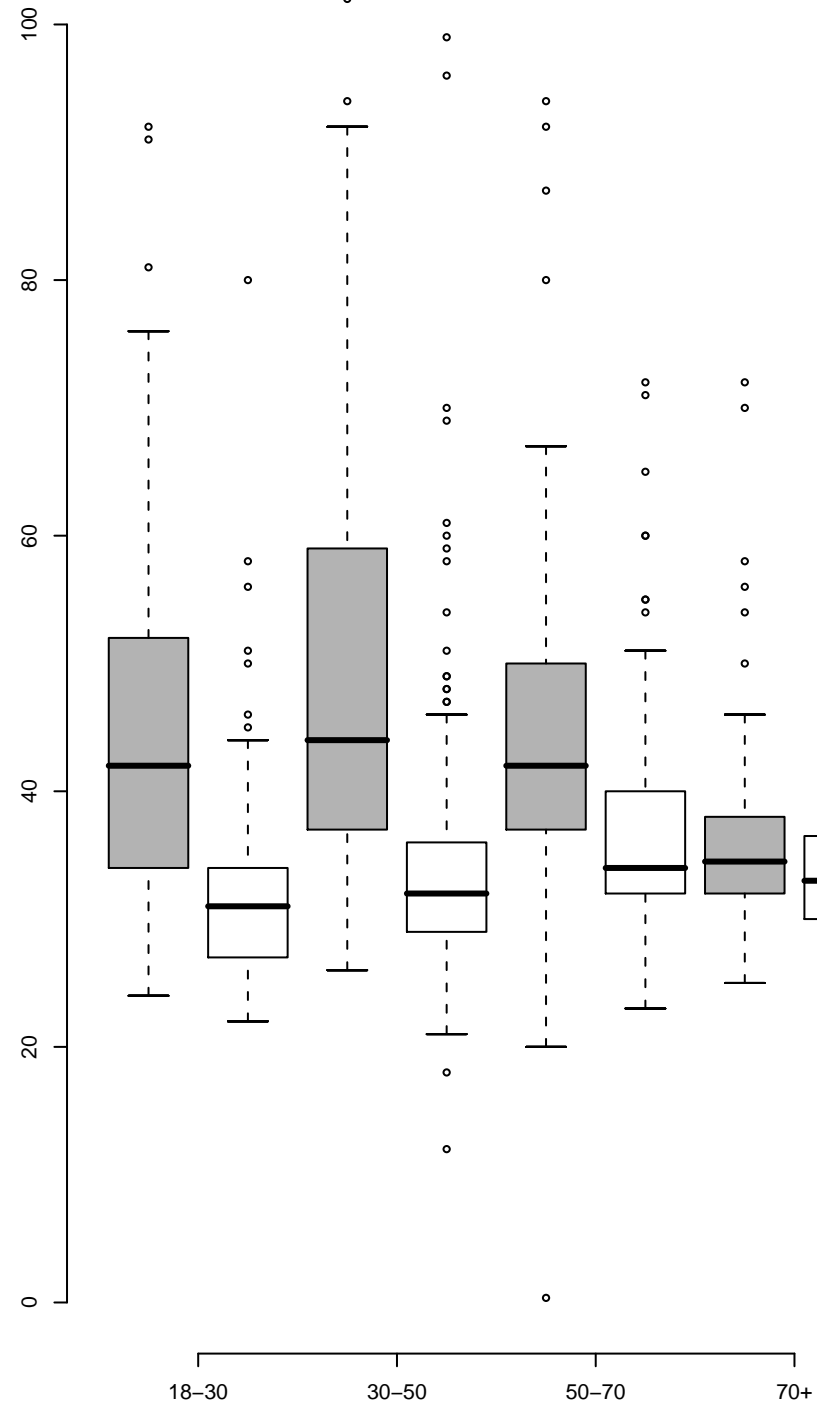

GGT (U/l)

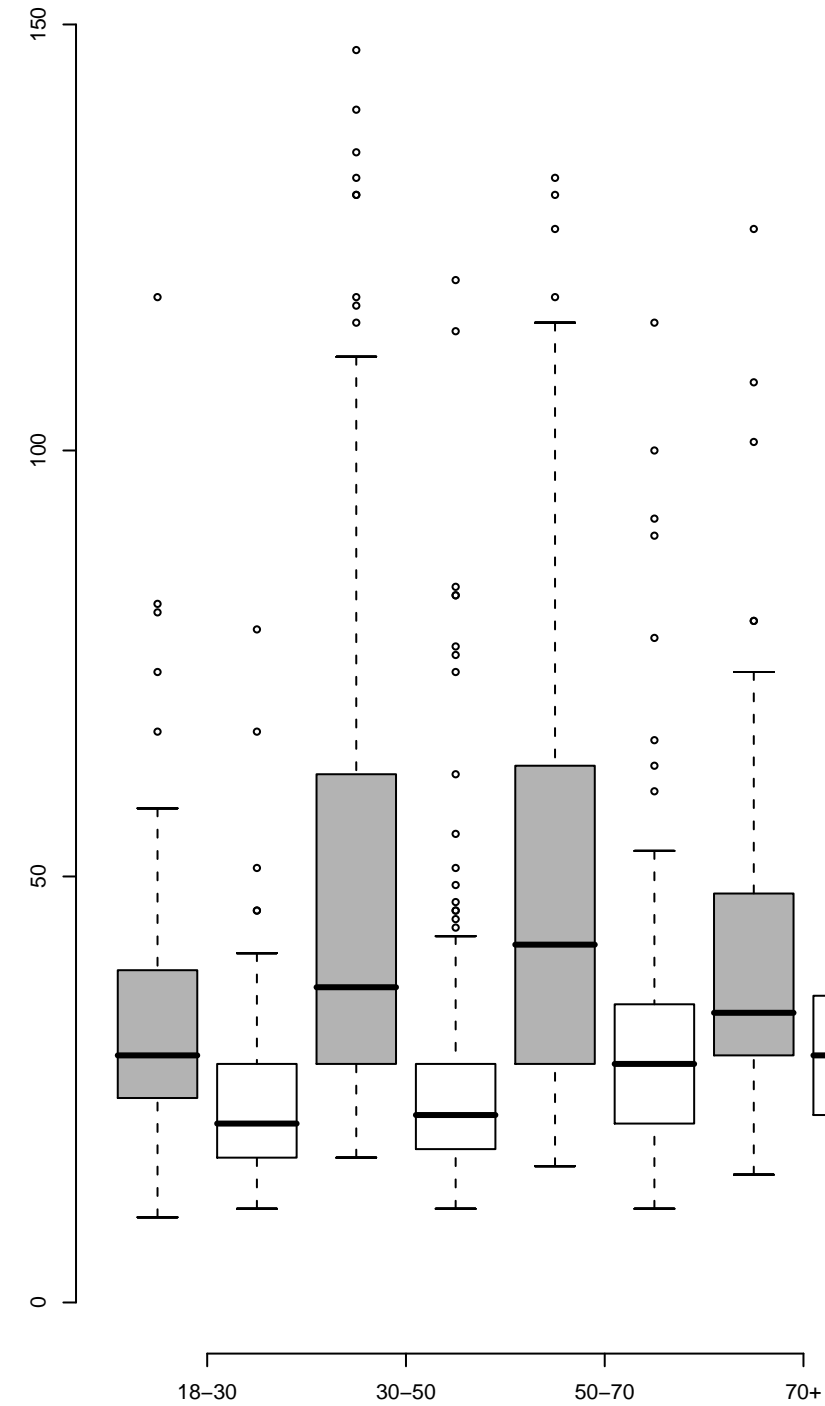

Supplement: Additional file 3 — Distribution of liver-related intermediate phenotypes. Distribution of glutamic-oxaloacetic transaminase (GOT), glutamate pyruvate transaminase (GPT) and gamma glutamyl-transpeptidase (GGT), by age and sex (gray = males, white = females). [file 1471-2350-8-29-S3.pdf]
